# Detection of *Mycobacterium tuberculosis* GlcB or HspX Antigens or *devR* DNA Impacts the Rapid Diagnosis of Tuberculous Meningitis in Children

**DOI:** 10.1371/journal.pone.0044630

**Published:** 2012-09-12

**Authors:** Sagarika Haldar, Naveen Sankhyan, Neera Sharma, Anjali Bansal, Vitul Jain, V. K. Gupta, Monica Juneja, Devendra Mishra, Arti Kapil, Urvashi B. Singh, Sheffali Gulati, Veena Kalra, Jaya Sivaswami Tyagi

**Affiliations:** 1 Department of Biotechnology, All India Institute of Medical Sciences, New Delhi, India; 2 Department of Pediatrics, All India Institute of Medical Sciences, New Delhi, India; 3 Department of Biochemistry and Department of Pediatrics, Dr. Ram Manohar Lohia Hospital, New Delhi, India; 4 Department of Pediatrics, Maulana Azad Medical College and Lok Nayak Hospital, New Delhi, India; 5 Department of Microbiology, All India Institute of Medical Sciences, New Delhi, India; McGill University, Canada

## Abstract

**Background:**

Tuberculous meningitis (TBM) is the most common form of neurotuberculosis and the fifth most common form of extrapulmonary TB. Early diagnosis and prompt treatment are the cornerstones of effective disease management. The accurate diagnosis of TBM poses a challenge due to an extensive differential diagnosis, low bacterial load and paucity of cerebrospinal fluid (CSF) especially in children.

**Methodology/Principal Findings:**

We describe the utility of ELISA and qPCR for the detection of *Mycobacterium tuberculosis* (*M. tb*) proteins (GlcB, HspX, MPT51, Ag85B and PstS1) and DNA for the rapid diagnosis of TBM. CSF filtrates (n = 532) derived from children were classified as ‘Definite’ TBM (*M. tb* culture positive, n = 29), ‘Probable and Possible’ TBM (n = 165) and ‘Not-TBM’ including other cases of meningitis or neurological disorders (n = 338). ROC curves were generated from ELISA and qPCR data of ‘Definite’ TBM and Non-Tuberculous infectious meningitis (NTIM) samples and cut-off values were derived to provide ≥95% specificity. *devR* qPCR, GlcB, HspX and PstS1 ELISAs showed 100% (88;100) sensitivity and 96–97% specificity in ‘Definite’ TBM samples. The application of these cut-offs to ‘Probable and Possible’ TBM groups yielded excellent sensitivity (98%, 94;99) and specificity (98%, 96;99) for qPCR and for GlcB, HspX and MPT51 antigen ELISAs (sensitivity 92–95% and specificity 93–96%). A test combination of qPCR with GlcB and HspX ELISAs accurately detected all TBM samples at a specificity of ∼90%. Logistic regression analysis indicated that these tests significantly added value to the currently used algorithms for TBM diagnosis.

**Conclusions:**

The detection of *M. tb* GlcB/HspX antigens/*devR* DNA in CSF is likely to improve the utility of existing algorithms for TBM diagnosis and also hasten the speed of diagnosis.

## Introduction

The global burden of tuberculosis (TB) is immense with an estimated incidence of 8.8 million cases and 1.45 million deaths in 2010 [1]. Tuberculous meningitis (TBM) is one of the most devastating manifestations of extrapulmonary tuberculosis (EPTB) with an estimated mortality of 1.5 per 100,00 population in India [2]. Prompt diagnosis is a necessity to reduce morbidity and mortality, especially in children [3], [4]. In addition, similar clinical or biochemical presentations in cases of partially treated pyogenic meningitis and other infectious and non-infectious neurological disorders often pose a challenge to the clinician. Therefore, accurate, rapid, inexpensive and simple tests are urgently needed for TBM diagnosis.

We earlier demonstrated that the diagnosis of TBM was enhanced by detecting DNA in CSF filtrates. In view of the high diagnostic accuracy of PCR (sensitivity ∼88%, specificity - 92%) in that study [5], *devR* DNA was quantified in CSF samples in the present study. However, the widespread implementation of nucleic acid amplification tests (NAATs) for TB diagnosis in resource-limited TB-endemic settings is hampered by the need for sophisticated instrumentation and technical expertise. In this context it was surmised that *M. tb* antigens in CSF filtrates could perhaps be exploited in the rapid diagnosis of TBM. Accordingly, five *M. tb* antigens *i.e.* GlcB, HspX, MPT51, Ag85B and PstS1 were quantified in CSF filtrates to evaluate their utility in the diagnosis of TBM. These antigens were selected for their expression in the early stages of TB infection independent of HIV co-infection, namely, GlcB and MPT51 [6], [7], or their secretory properties, namely, Ag85B and PstS1 [8], [9], or being associated with cavitary disease such as PstS1 [10], [11], or being expressed *in vivo*, such as HspX [12], or their use in TBM diagnosis, eg. Ag85B and HspX [13]–[15]. Notably, four of these five antigens (GlcB, MPT51, PstS1 and Ag85B) were amongst 20 *M. tb* culture filtrate antigens that elicited a robust antibody response, implying that these antigens were expressed during active disease and were immunogenic [16]. HspX and PstS1 antigens were also found to be amongst the proteins targeted by antibodies in patient sera from active TB patients [17].

CSF specimens were categorized according to the uniform case definition rule defined recently that is based on clinical criteria, CSF parameters, CT findings and presence of extraneural TB [18]. Logistic regression analysis revealed that antigen/qPCR test results significantly enhanced the utility of existing diagnostic algorithm for TBM diagnosis when considered along with the case definition for TBM (p<0.0001). Our results demonstrate that antigen/DNA detection hold promise for the development of rapid tests for TBM diagnosis.

## Materials and Methods

### Objectives

This study was primarily designed to (i) to quantify *M. tb* GlcB, HspX, MPT51, Ag85B and PstS1 proteins and DNA in CSF filtrates for the diagnosis of childhood TBM, (ii) to compare the performance of antigen and DNA detection tests, and (iii) to assess the impact of these tests on the standard diagnosis of TBM.

### Ethics Statement

Ethical clearance to collect CSF samples was taken from the Institutional Ethics committee of Dr. Ram Manohar Lohia Hospital (RML), Ethics committee of All India Institute of Medical Sciences (AIIMS) and the Institutional Ethics committee of Lok Nayak Hospital (LNH). Since the study was conducted on children, all samples were collected after obtaining a written informed consent from parents of the children. Individual patient data was collected using a questionnaire (Appendix S1). All data were treated in a strictly confidential manner following the ethical principles of the Helsinki Declaration of 1964 revised by the 59th WMA General Assembly, Seoul in October 2008.

### Participants

Five hundred and fifty five CSF samples were collected consecutively for this prospective, cross sectional study between April 2007 and April 2010 from pediatric wards of RML, AIIMS and LNH, New Delhi. The clinical data of these subjects was collected simultaneously. CSF samples were collected from children aged <1 month to 17 yrs and CSF from children with known or suspected malignancies and those on antituberculosis chemotherapy (ATT) >14 days were excluded from the study. Five hundred and thirty two samples of the 555 samples were finally included in the analysis; 23 samples were excluded as no clinical data was available for them. The biochemical, cytological and radiological investigations were performed at RML, AIIMS and LNH and microbiological, ELISA and PCR analysis were performed at AIIMS. For microbiological, ELISA and PCR analysis, CSF aliquots were transported to AIIMS within 12–36 hours and processed. All the laboratory personnel were blinded to clinical information, diagnosis and the results of other laboratory investigations. All the results were analyzed upon completion of the study.

### Definition and Classification of CSF Samples

**Figure 1:**
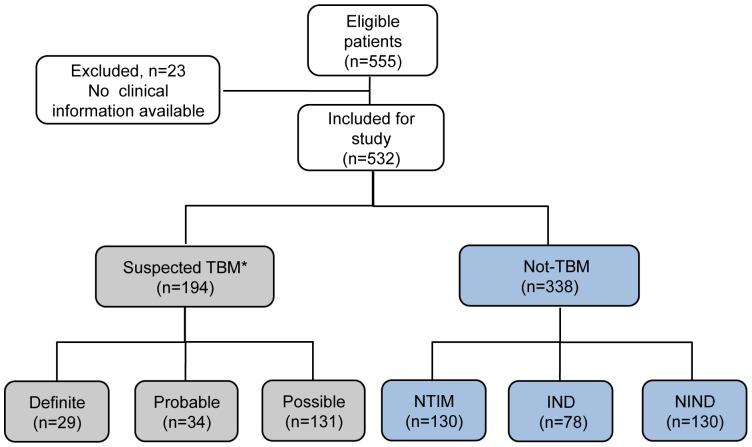
Categorization of CSF samples. *Suspected TBM were classified into ‘Definite’, ‘Probable’ and ‘Possible’ categories; ‘Not-TBM’ included Non Tuberculous infectious meningitis (NTIM), Infectious neurological disorders (IND) and Non-infectious neurological disorders.

After all the assays were completed, the CSF samples were categorized according to recently defined criteria [18] by which ‘Definite’ TBM includes culture/smear/commercial NAAT positive/AFB seen on autopsy; ‘Probable and Possible’ TBM groups include subjects negative by the above criteria but satisfying defined clinical criteria, CSF criteria, cerebral imaging criteria and evidence of extraneural TB having a score of ≥10–12 (‘Probable’ TBM) and a score of ≥6–11 (‘Possible’ TBM). In our study, samples were classified as ‘Definite’ TBM on the basis of *M. tb* culture positivity only (n = 29), as ‘Probable’ TBM (n = 34, score range: 10–18), ‘Possible’ TBM (n = 131, score range: 6–9) and ‘Not-tuberculous’ meningitis with an alternative diagnosis established (n = 338). The ‘Not-TBM’ category was further sub-divided into Non-tuberculous infectious meningitis (NTIM, n = 130), Infectious neurological disorders (IND, n = 78) and Non-infectious neurological disorders (NIND, n = 130) (Figure 1). The median age and range of the children in each diagnostic category were quite comparable (Table S1). The NTIM comprised of cases of pyogenic bacterial meningitis that included culture confirmed cases of group B streptococci (n = 2), *E. coli* (n = 2), *Staphylococcus aureus* (n = 5), *S. aureus* (MRSA, n = 2), *Pseudomonas sp* (n = 2), *Enterococcus sp* (n = 4), *Klebsiella sp* (n = 1) and *Acinetobacter sp* (n = 7). Other cases were diagnosed on the basis of response to appropriate antibiotics, clinical presentation and symptoms. The IND category included 30 cases of meningoencephalitis, 14 cases of sepsis, 9 cases each of enteric encephalopathy and aseptic meningitis, 5 cases each of cerebral malaria and pneumonia, 1 case each of dengue fever, acute gastroenteritis, post diphtheritic polyneuritis, tetanus, urinary tract infection and viral gastritis. The NIND category included 30 cases of neurodegenerative disorders, 24 cases of seizure disorders, 23 cases of Guillain–Barré syndrome, 17 cases of febrile seizures, 8 cases of mitochondrial cytopathy, 3 cases each of hypocalcemic seizures and febrile seizures, 2 cases each of acute diarrheal disease, transverse myelitis, congenital hydrocephalous, intracranial space occupying lesion, drug induced rash, head injury, hemiparesis, myopathy, post traumatic seizures, spinal cord compression and rickets.

### Specimen Processing and DNA Isolation

CSF (0.5 ml –3 ml) was filtered through a 0.22 µm PVDF membrane filter (Millipore) to obtain ‘filtrate’ and ‘sediment’ fractions. The entire membrane (sediment fraction) was transferred to Middlebrook 7H9 media (BD) supplemented with Albumin Dextrose complex (ADC) and PANTA (BD) and incubated at 37°C for up to 4 weeks. A minimum of 0.5 ml of whole CSF was also inoculated into BD BACTEC™ MGIT™ 960 system with OADC according to manufacturer’s instructions. Approximately 0.5 ml filtrate aliquots were processed for DNA isolation as described [5] and used for PCR. The remaining untreated filtrate fractions were stored at −80°C for ELISA.

### PCR

DNAs of all CSF samples were analyzed by qPCR and *devR-*IS*6110* duplex PCR (gel-based detection). The qPCR assay amplified a 144-bp region of *devR* using primers devRf4 and devRr3 [5]. Reactions contained 1×Power SYBR Green Master Mix (Applied Biosystems), 0.5 µM of each primer and specimen DNA in a volume of 25 µl. A standard curve was generated alongside to quantitate the bacterial DNA load in each sample.

A duplex PCR targeting 308-bp sequence of *devR* and 123-bp of IS*6110* was developed using devRf2/devRr2 and T4/T5 primer pairs [19]. The reactions contained 0.5 µM each of forward and reverse primers, 1×PCR buffer, 2 mM MgCl_2_, 0.25 mM dNTPs and 1.5 U Taq DNA polymerase. The tubes were subjected to 10 min at 94°C, 45 cycles each of 1 min at 94°C, 45 sec at 59°C, 45 sec at 72°C and finally 7 min at 72°C. A positive control having *M. tb* DNA and 2 negative control reactions lacking DNA were always included. The amplification products were detected by ethidium bromide staining and visualization under UV light after electrophoresis on a 2.3% agarose gel. The amplification of either target was considered to be positive. The absence of PCR inhibitors was established by inhibitor check reactions that were spiked with *M. tb* DNA.

### Protein Purification

Recombinant bacterial expression plasmids encoding hexahistidine fusions of M. tb GlcB (Rv1837c), HspX (Rv2031c), MPT51 (Rv3803c), Ag85B (Rv1886c) and PstS1 (Rv0934) proteins were provided by the Tuberculosis Research Materials and Vaccine Testing Contract at Colorado State University. These proteins were purified by Ni- NTA affinity chromatography using standard techniques using ÄKTA™ FPLC system to ≥90% purity (GE Healthcare, USA). HspX was purified as described [12]. The purified proteins were used to raise polyclonal antiserum in rabbits according to the immunization schedule described earlier [20]. The antibodies were generated in the Central Animal Facility at AIIMS after obtaining due ethical clearance from the Institutional Animal Ethics Committee, AIIMS. IgG antibodies for ELISA were purified using Melon™ gel IgG spin purification kit (Pierce, USA).

### Indirect ELISA for *M. tb* Antigen Detection

A standard curve was generated using two-fold serial dilutions of the recombinant *M. tb* proteins in neat and spiked CSF (1–250 ng/well). For antigen detection, triplicate wells in a 96-well microtiter plate were coated with each CSF sample (100 µl of 1∶20 dilution) and incubated overnight at 4°C. The plates were blocked with 3% BSA in phosphate buffered saline (PBS), thereafter polyvalent antibody (100 µl of 1∶2500 dilution) against individual *M. tb* antigens was added to sample wells and the plates were incubated at 37°C for 2 hrs. The wells were washed with PBS - 0.2% Tween 20 and with PBS followed by the addition of the secondary antibody (goat anti-rabbit IgG-HRP, Bangalore Genei, Bengaluru, India) and incubation for one hour at 37°C. The wells were again washed as above and substrate o-Phenylene diamine (OPD, 0.5 mg/ml in citrate phosphate buffer; Sigma Aldrich, USA) was added to each well and the absorbance was measured at 490 nm. Appropriate ELISA controls were included in each assay.

### Statistical Analysis

Cytology and biochemical features of CSF from TBM (‘Definite’, ‘Probable’ and ‘Possible’) and ‘Not-TBM’ (NTIM, IND, NIND) patients were statistically analyzed using Mann Whitney U test. To evaluate the diagnostic potential of the DNA and antigen detection tests, DNA amounts and ΔOD_490_ values in CSF from ‘Definite’ TBM (true positives) and NTIM group (true negatives) were used to construct ROC curves using (GraphPad Prism version 5.00 for Windows, GraphPad Software, San Diego California USA, www.graphpad.com). The cut-off points were derived to achieve test specificity (≥95%) consistent with recent recommendations for diagnostic tests targeting childhood TB [21]. These cut-off points were then applied to the ‘Probable and Possible’ TBM, IND and the NIND groups of subjects to determine assay performance.

ELISA and PCR performance parameters such as sensitivity, specificity, positive predictive value, negative predictive value, positive and negative likelihood ratios were calculated using resources at http://ktclearinghouse.ca/cebm/practise/ca/calculators/statscalc link provided on the http://www.teachepi.org website.

In order to examine additional benefit of these ‘new’ tests we excluded the ‘Definite’ TBM group and generated a logistic regression model with the remaining samples to assess whether they (ELISA and qPCR) provided incremental advantage in diagnosing ‘Probable and Possible’ TBM cases over and above the defined diagnostic algorithm [18]. For this analysis, eighteen co-variates were considered, namely, clinical, CSF and cerebral imaging criteria and evidence of extraneural TB (fever and headache >5 days, weight loss, history of contact, high cell count, lymphocyte predominance, low CSF to blood sugar ratio, raised amount of proteins and positive CT findings, etc.). This model estimated the log odds of the disease probability as a function of these 18 predictors. The ability of this model to discriminate between patients with and without TBM was estimated using the area under the ROC curve. Logistic Regression analysis was performed using STATA 8.0 software.

## Results

CSF samples were collected from 555 children from three centers in New Delhi, India. Out of these, results of 532 samples were available and they were classified as TBM (n = 194); Non tuberculous infectious meningitis, NTIM (n = 130); Infectious neurological disorders, IND (n = 78); and Non infectious neurological disorders, NIND (n = 130). ATT was administered to all 194 patients of suspected tuberculous meningitis. *M. tb* was isolated from the CSF of 29 patients (Definite TBM), and the rest were defined as having ‘Probable and Possible’ TBM. The majority of TBM cases (∼95%) presented with an advanced TBM disease (British Medical Research Council [BMRC] of grade 2 or 3) [22]; 101 patients were classified as BMRC grade 2 at presentation, 83 as grade 3, and 10 as grade 1. All Definite TBM subjects belonged to either BMRC grade 2 or 3. Outcomes for response to ATT were available in 178 of the 194 children with TBM. Of these, 27 children died and remaining 151 children showed response to therapy on follow up. All the 532 specimens were subjected to biochemical analysis, cytology examination, culture, ELISA and PCR.

### Conventional Test Results

Twenty nine CSF samples were positive for *M. tb* by liquid culture technique (in-house liquid culture/BACTEC MGIT). Smear microscopy was not performed as smear positivity in CSF specimens from pediatric subjects was observed to be nil in an earlier study [5], [23] and the filters were directly inoculated into culture medium [24]. None of TBM samples (n = 194) were positive for any other bacterial pathogen. The total cell count was significantly higher in the TBM group *vs.* the IND and NIND group (p<0.0001, Table S1). However, the TBM group showed a predominantly lymphocytic reaction coupled with a significantly lesser polymorphonuclear cell response (neutrophils) *vs.* the NTIM group (Table S1). Forty eight percent of the TBM patients had protein levels >100 mg/dl in contrast to only 29% of the NTIM group (Table S1). The difference in the CSF to blood sugar ratio in the TBM and other groups was also statistically significant (Table S1). Thus the cytological and biochemical test results were consistent with previous findings [25].

Amongst children with TBM, only 19% had a prior history of contact with a TB patient and symptoms such as fever, headache and vomiting were not discriminatory across various groups. The presence of hydrocephalus (47%) and basal exudates (29%) strongly favored a diagnosis of TBM. A positive Mantoux test was seen in 38% of the children with TBM as compared to 1–2% of the control group. BCG vaccination status did not differ significantly amongst all the groups and therefore did not appear to be protective in this study. Thirty-one children (11%) in the TBM category had associated extraneural TB (Pulmonary TB-17, Miliary/Disseminated TB-7, Abdominal TB-4, Lymph Node TB- 2, Gastric TB-1, Figure S1).

### DNA Detection Test Results

**Figure 2:**
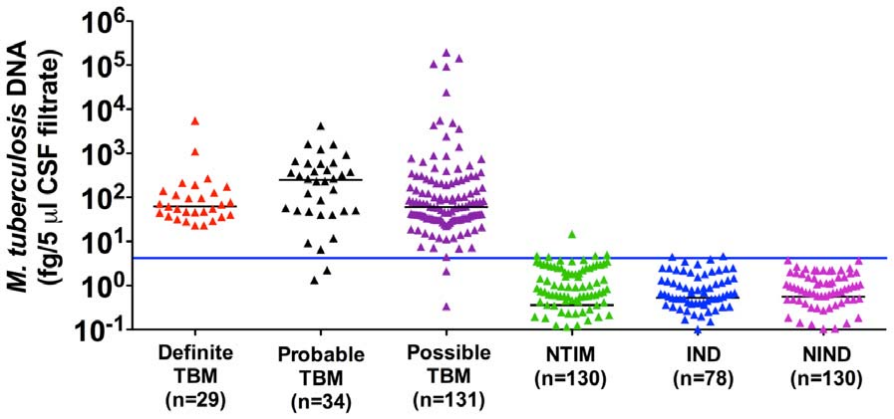
DNA quantitation in CSF filtrates by qPCR. Scatter plot of starting bacterial DNA load (in 5 µl of CSF). The horizontal bar denotes the median value (black line in individual data sets) and the cut-off points (blue line across the plot). The cut-off points were determined by ROC curve analysis of DNA amounts in CSF from ‘Definite’ TBM (true positives) and NTIM (true negatives) groups.

DNA was quantitated by qPCR and ranged from 1 bacterial genome equivalent to 1.57×10^5^ bacterial genome equivalents (Mean±SD, 2584.7±16096.3) per 5 µl of CSF in the TBM group (Figure 2, Figure S2). DNA amounts in CSF from ‘Definite’ TBM (true positives) and NTIM groups (true negatives) were used to construct ROC curves and the cut-off value was set at ∼4 fg *M. tb* DNA (∼1 *M. tb* genome equivalent) to achieve a specificity of 96% (95% CI: 91, 99). qPCR performed exceedingly well and yielded a sensitivity of 100% (95% CI: 88, 100; Table 1, Figure 2). A similar sensitivity of 98% (95% CI: 94, 99) and a specificity of 98% (95% CI: 96, 99) was also attained in ‘Probable and Possible’ TBM groups (Table S2).

**Table 1:** Performance of ELISA and PCR assays in Definite TBM subjects.^a^

PCR/ELISA	Sensitivity^b^	Specificity^b^	PPV^b^	NPV^b^	LR+	LR−
qPCR	100 (88;100)	96 (91;99)	84 (69;93)	99 (96;100)	23 (10;53)	0.02 (0;0.27)
GlcB	100 (88;100)	96(91;99)	84 (69;93)	99 (96,100)	23 (10;53)	0.02 (0;0.27)
HspX	100 (88;100)	96 (91;99)	84 (69;93)	99 (96;100)	23 (10;53)	0.02 (0;0.27)
MPT 51	90 (73;98)	98(95;100)	93 (77;98)	98 (93;99)	58 (14;232)	0.11 (0.04;0.31)
Ag 85B	79 (60;92)	97 (92;99)	85 (67;94)	95 (90;98)	26 (10;68)	0.21 (0.1;0.44)
PstS1	100 (88;100)	97 (92;99)	87 (72;94)	99 (96;100)	29 (11;71)	0.02 (0;0.27)

a using cut-offs determined from ROC analysis of ELISA/qPCR data from ‘Definite’ TBM and NTIM groups (true positives and true negatives, respectively).

b Values are in percentages, values in brackets denote 95% confidence intervals.

Duplex PCR had a sensitivity of 66% (95% CI: 47, 80) and specificity of 93% (95% CI: 86, 96) in the ‘Definite’ TBM group as compared to 83% sensitivity in the ‘Probable and Possible’ TBM group with similar specificity of 93% (95% CI: 89, 95; Table S3). None of the samples positive in the Duplex PCR were negative for the IS*6110* target.

### Antigen Detection Test Results

**Figure 3:**
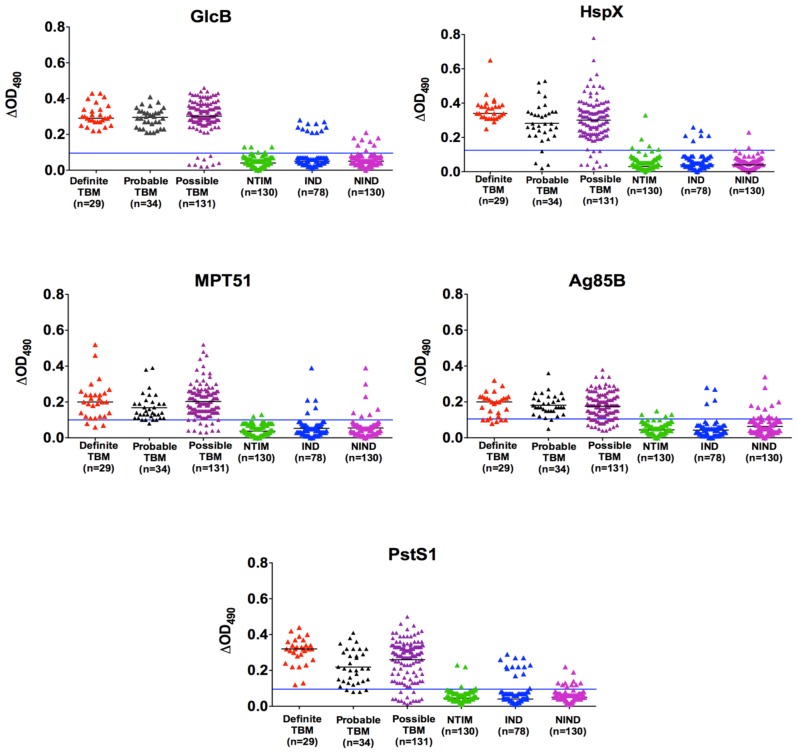
Antigen detection in CSF filtrates by ELISA. Scatter plots of GlcB, HspX, MPT51, Ag85B and PstS1 antigens (in 5 µl of CSF) showing ΔOD values. Using ROC curves generated from ΔOD values in CSF of ‘Definite’ TBM and NTIM group, ΔOD cut-off values at 0.095, 0.125, 0.1, 0.105 and 0.095 were selected for GlcB, HspX, MPT51, Ag85B and PstS1, respectively. The horizontal bar denotes the median value (black line in individual data sets) and the cut-off points (blue line across the plots).

Standard curves were generated for GlcB, HspX, MPT51, Ag85B and PstS1-spiked CSF by indirect ELISA (Figure S2). ΔOD_490_ values obtained with CSF from ‘Definite’ TBM (true positives) and NTIM groups (true negatives) were used to construct ROC curves and cut-off values were set as indicated in Figure 3 for GlcB, HspX, MPT51, Ag85B and PstS1, respectively. Comparison of the ‘Definite’ TBM group with the NTIM group yielded a sensitivity of 100% (95% CI: 88, 100) at ≥96% specificity for GlcB, HspX and PstS1 ELISAs (Table 1). Ag85B and MPT51 ELISAs had a comparatively lower sensitivity of 79% (95% CI: 60, 92) and 90% (95% CI: 73, 98), respectively with a specificity of ≥97%. GlcB, HspX and MPT51 were the most valuable antigens in the Probable and Possible’ TBM groups with a sensitivity range of 92–95%. Ag85B and PstS1 assays had a somewhat lower sensitivity of 84% (95% CI: 77, 89) and 89% (95% CI: 83, 93), respectively. All assays had a specificity ranging from 92–96% (Table S2).

### Comparison of PCR and ELISA Test Outcomes in Definite TBM

The performance characteristics of all the tests in ‘Definite’ TBM samples are shown in Table 1. qPCR assay had a LR+ of 23 and a LR− of 0.02. Amongst the ELISA-based assays, HspX and GlcB demonstrated similar values. MPT51, Ag85B and PstS1 assays had a higher LR+ albeit with a lower LR−. The high predictive accuracy of qPCR, GlcB and HspX tests suggests their utility for ruling in as well as ruling out the occurrence of TBM in children.

### Logistic Regression Analysis Demonstrates Value Addition by ELISA and qPCR in TBM Diagnosis

**Figure 4:**
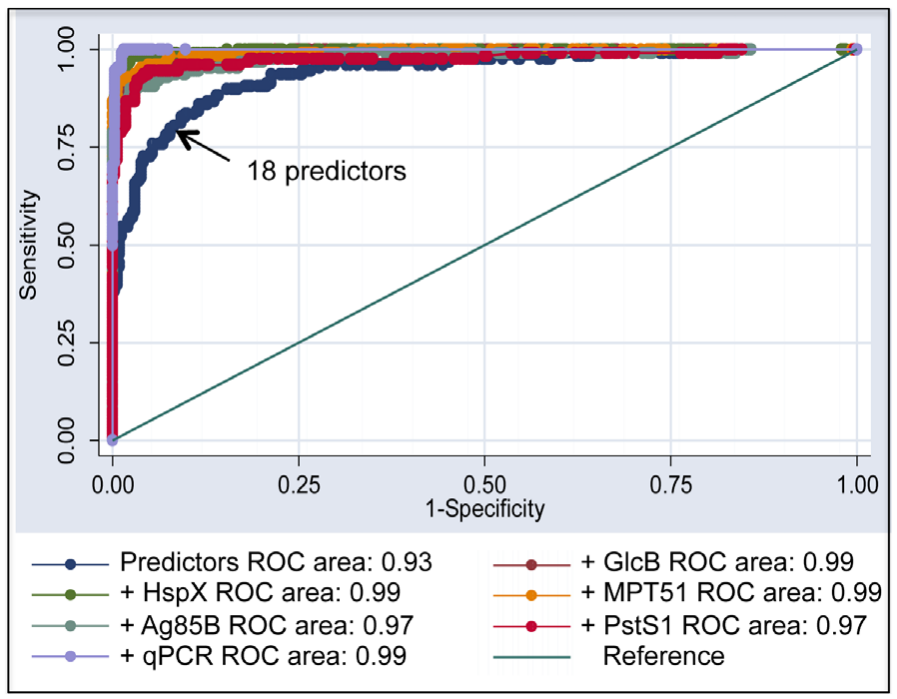
ROC curves for logistic regression analysis of ‘Probable and Possible’ TBM samples. Predictor model (18 predictors) applied to ‘Probable and Possible’ TBM combined with ELISA/qPCR.

Due to the poor yield of microbiological tests, multiple diagnostic determinants, namely, medical history, clinical signs and symptoms, biochemical and cytological analysis as well as imaging techniques are applied for the diagnosis of TBM. All the data obtained in the present study (Figure S1 and Table S1) was used to stratify patients as ‘Definite’ (*M. tb* culture positive), ‘Probable and Possible’ TBM and ’Not-TBM’, based on a consensus case definition for the diagnosis of TBM [18]. The additional benefit of ELISA and qPCR was evaluated by excluding from the cohort ‘Definite’ TBM samples and generating a logistic regression model that determined whether the ‘new’ tests provided incremental advantage in diagnosing TBM over and above the defined diagnostic algorithm. This algorithm [18] that comprised of 18 predictors was considered as a “single” test and its ROC curve was generated (Figure 4). Amongst the various predictors, the most valuable determinants (p<0.05) in this study were presence of fever, headache, weight loss, history of contact, high cell count, lymphocyte predominance, low CSF to blood sugar ratio, raised amount of proteins and positive CT findings (hydrocephalus and basal exudates, Table S4). A highly significant increase in area under the ROC curve from ∼0.944 to >0.97 (p<0.0001) was noted upon addition of the ‘new’ tests and thereby established their positive impact on TBM diagnosis (Figure 4). Moreover these tests are rapid and thus, are likely to hasten the accurate diagnosis of TBM.

## Discussion

In the present study, we evaluated the utility of *M. tb* antigen and DNA detection in CSF for the rapid and accurate diagnosis of TBM. We believe this to be the first study to simultaneously quantitate *M. tb* protein antigen and DNA in CSF for TBM diagnosis.

### Heterogeneous *M. tb* Populations Co-exist in CSF

The detection of HspX ‘dormancy antigen’ in CSF from patients with active disease was striking because its expression is believed to be induced upon bacterial exposure to dormancy-associated signals [26]. In contrast, anti-HspX antibodies were detected in asymptomatic TB contacts rather than in those with active disease [27]. Moreover, the occurrence of mycobacterial heterogeneity and dormant bacterial sub-populations in active TB patients and animal models has been reported [28], [29]. Therefore the efficient detection of HspX antigen suggests that the bacteria may be exposed to dormancy-inducing signals in the CSF milieu.

The detection of other antigens in CSF is also noteworthy. GlcB and MPT51 are believed to be expressed very early during infection [6], while PstS1 and Ag85B are associated primarily with multibacillary or advanced disease [23], [30]. Interestingly, the arrest of *M. tb* multiplication in mouse lung is accompanied by a significant decrease in the levels of Ag85 complex- and PstS1- encoding mRNAs [31]. The detection of ‘dormancy’ antigen on one hand and secretory proteins and those involved in cell wall synthesis, cell adhesion and metabolism on the other hand in the CSF suggests that bacteria of varying and diverse physiological states and phenotypes may coexist in the CSF environment.

### Active Bacterial Lysis in CSF

Immunopathogenesis during TBM is reportedly associated with elevated levels of TNF-α and the upregulation of genes such as IL1B, IL12B, TNF etc. which are usually involved in host inflammatory responses [32]–[34]. The release/presence of bacterial components in CSF filtrates may be attributed to an extensive immune response. The presence of diverse antigens suggests that lysis is not targeted to either actively replicating or ‘dormant’ bacteria. However, regardless of the pathophysiological mechanisms in play, from the diagnostic standpoint, the detection in CSF of diverse *M. tb* antigens establishes the utility of ELISA assays for TBM diagnosis.

### Antigen Concentrations in Various TBM Sub-groups

**Figure 5:**
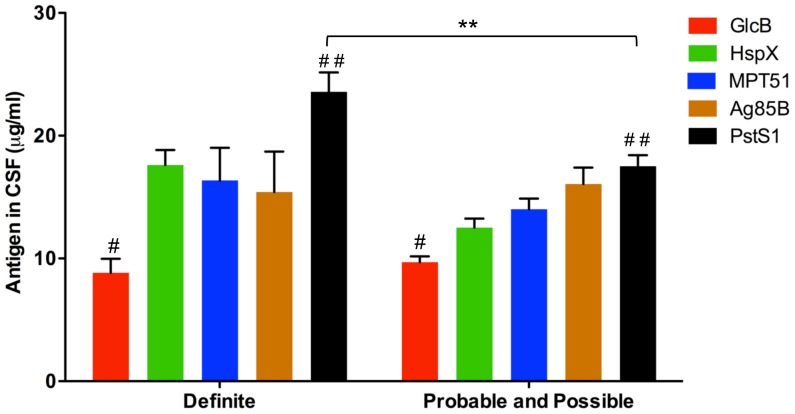
Antigen concentrations in TBM CSF filtrates. ^#^GlcB concentration was significantly lower (p<0.05) than that of HspX, MPT51 and PstS1 in ‘Definite’ TBM and all 4 antigens in ‘Probable and Possible’ TBM groups; ^##^PstS1 concentration was significantly higher (p<0.05) within both ‘Definite’ (all antigens) and ‘Probable and Possible’ TBM (all antigens except Ag85B) groups. **PstS1 concentration was also significantly higher (p<0.01) in ‘Definite’ TBM group *vs.* ‘Probable and Possible’ TBM groups.

A comparative analysis revealed PstS1 antigen concentration to be significantly higher than other antigens in the ‘Definite’ TBM group (Figure 5). This is consistent with PstS1 antigen being a major culture filtrate protein [9]. It is noteworthy that although the concentration of GlcB in CSF was significantly lower than that of the other antigens tested in all TBM groups, GlcB ELISA showed excellent sensitivity and specificity, indicating its value as a diagnostic antigen (Table 1 and Table S2). The levels of GlcB, MPT51 and Ag85B did not differ significantly in ‘Definite’ *vs.* ‘Probable and Possible’ TBM groups, however HspX and PstS1 were significantly lower in the latter group (Figure 5).

### Protein *vs.* DNA Detection

**Figure 6:**
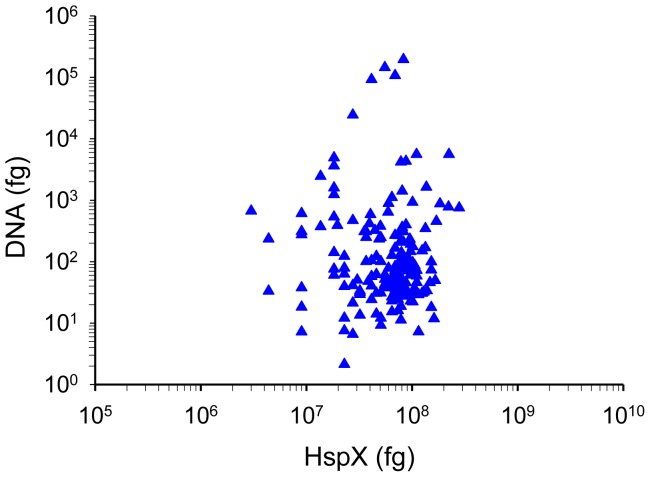
Comparison of *M. tb* DNA *vs.* antigen amount in CSF. A representative graph of HspX is shown. All other antigens showed a similar pattern (Figure S3). Starting amounts of DNA and antigen in 5 µl CSF were quantitated by qPCR and ELISA, respectively.

Towards assessing the relative advantage of the diagnostic assays, DNA and antigen amounts (fg) were estimated in CSF filtrates (Figure 6). The mean load of HspX exceeded that of DNA by ∼thousand to million- fold (HspX antigen mean load, 6.64×10^7^±4.6×10^7^ fg *vs.* DNA load, 3.23×10^3^±2.01×10^4 ^fg) and supports the utility of antigen detection *vs.* DNA detection in CSF (GlcB, MPT51, Ag85B and PstS1 also showed a similar skew in load *vs.* DNA, Figure S3). The accurate and direct detection of antigen in very small volumes of CSF (5 µl) is a crucial practical factor in favor of antigen detection whereas DNA-based tests much larger volumes of CSF (minimum 100–500 µl).

### False Positive and False Negative Results

Four to 8% false positivity was observed in the antigen ELISAs (Table 1 and Table S2) and included samples with diagnosis of pyogenic bacterial meningitis, meningoencephalitis, cerebral malaria, sepsis, aseptic meningitis, urinary tract infection and dengue fever. Six parameters that are predictive of TBM, namely, fever >5days, altered consciousness, high cell count, high protein concentration, low CSF to blood sugar ratios and lymphocytic predominance, were also positive in those NTIM and IND samples called positive by the ‘new’ tests. The overlap of these features between TBM, NTIM and IND groups may be due to an infectious etiology. In the NIND group (cases of transverse myelitis, seizures, Guillain–Barré syndrome, mitochondrial cytopathy), these parameters were generally negative except in the case of transverse myelitis. Protein BLAST analysis did not reveal the presence of homologues to these antigens amongst the potential pathogens associated with meningitis. A possible reason for false positives maybe the use of polyclonal antisera that cross-react with common epitopes. Some false positives were common for MPT51 and Ag85B detection, which can be attributed to ∼40% identity in their sequences [35]. This is consistent with a reported false positivity of ∼13% for Ag85 complex in TBM diagnosis [13].

Despite high assay sensitivities, a few positive samples were missed by the ELISAs. False negatives may be attributed to either low antigen concentration in paucibacillary disease or antigen sequestering within antigen-antibody complexes. GlcB, HspX and PstS1 ELISAs did not miss any sample in the ‘Definite’ TBM category and GlcB antigen was missed only in samples of the ‘Possible’ TBM group. Despite sequence homology between MPT51 and Ag85B, the sensitivity of MPT51 detection was uniformly higher than that of Ag85B in TBM samples (Table 1 and Table S2). The better performance of the former could be attributed to the presence of specific sequences in its C-terminal domain [36]. PstS1 ELISA had a false negativity of 11% in ‘Probable and Possible’ TBM groups despite its high expression level (Figure 5). This is consistent with PstS1 concentration being significantly lower (p<0.01) in these groups as compared to the ‘Definite’ TBM group (Figure 5). Even so, the sensitivity of PstS1 antigen ELISA (89%) was superior to that of PstS1 antibody ELISA (47%) in smear positive pulmonary TB (PTB) patients [37] suggesting the superiority of antigen detection over antibody detection.

Based on a false positivity and false negativity of ∼2% each, the qPCR assay performance was judged to be very satisfactory (Figure 2, Table 1 and Table S2). While every sample of the ‘Definite’ TBM category was positive by qPCR and by one or more of antigen ELISAs, 4 samples in the ‘Probable and Possible’ TBM groups were missed by qPCR but positive by one or more ELISAs. Therefore the use of a combination of qPCR and two antigen ELISAs detected all TBM samples without lowering the specificity adversely (specificity- 90%) suggesting that a combinatorial approach is likely to be more accurate for disease diagnosis.

Twenty five of 194 children were on ATT (≤13 days) at the time of CSF sampling and included 15 children who received ATT >3 days all of which were positive by ELISA while only 1 sample was negative by qPCR. Thus, prior ATT >3 days did not appear to adversely affect the sensitivity of the ‘new’ tests.

### Strengths and Weaknesses of the Study

The major strength of this study is the development of laboratory tool(s) that improve the utility of the existing diagnostic algorithm for TBM and also hasten the speed of diagnosis. The highlights of the study are (i) *M. tb* proteins and DNA from lysed bacteria can be rapidly detected with a high degree of accuracy, (ii) antigen detection ELISAs have the potential to be simplified into a point-of-care format such as lateral flow immunochromatographic strip test, (iii) the qPCR assay has a utility in specialized laboratories, (iv) the ‘new’ tests appear to have a robust “rule-in rule-out” value. The excellent positive predictive value and positive likelihood ratios suggest using these tests to rule-in TBM diagnosis in cases of TBM. Likewise, the excellent negative predictive value and negative likelihood ratios suggest using the ‘new’ tests to rule-out TBM.

Our study also had some limitations. First, since cytology and biochemical testing was center-based there is an inherent variability in how CSF samples were analyzed and how other ancillary investigations were carried out. Second, although the CSF samples were processed within 12–36 hours in this study, we cannot exclude the possibility that delay in PCR or ELISA testing may have influenced test outcomes. The application of the ‘new’ tests to other extrapulmonary samples and to sputum may provide valuable insights into the diversity and dynamics of *M. tb* adaptation during infection. This study demonstrated that application of the ‘new’ ELISA and PCR tests improved the utility of the existing diagnostic algorithm used for the diagnosis of TBM. Although, WHO has strongly recommended against the use of current, commercial serodiagnostic tests for PTB or EPTB [38], meta-analysis of non-commercial antigen/antibody detection tests and antigen discovery–related proteomic studies have identified several candidate antigens/antigen combinations potentially useful for PTB serological test [16], [17], [37], [39]. Our data suggests that antigen(s) detection is an attractive diagnostic modality for point-of-care tests as it requires a tiny volume of sample, without a processing step and is not dependent on sophisticated equipment and has the potential to provide reliable, rapid and inexpensive diagnosis of TBM under conditions encountered commonly in developing countries such as India.

## Supporting Information

Figure S1 **Clinical, radiological and supportive findings.** Findings in TBM, NTIM, IND and NIND groups are shown. **A.** Clinical findings **B.** Radiological features **C.** Supportive findings.
(TIF)

Figure S2 **Standard curves for** ***devR*** **qPCR and** ***M. tb*** **antigen ELISAs.** *devR* qPCR standard curve was generated using *M. tb* DNA over a range of 5 fg to 5×10^7 ^fg (from 8 independent experiments, Mean±SD). Ct (threshold cycle) *vs.* logarithm of the amount of *M. tb* genomic DNA (fg) at the start of the reaction. ELISA standard curves for *M. tb* antigens were generated using purified proteins and spiked CSF. ELISA plates were coated with 2-fold serial dilutions of antigen ranging from 256 ng to 1 ng/well. The standard curves were used to quantitate antigen amounts in CSF.
(TIF)

Figure S3 **Comparison of** ***M. tb*** **DNA** ***vs.*** **antigen amount in CSF.** Graphs for GlcB, MPT51, Ag85B and PstS1 are shown. Starting amounts of DNA and antigen in 5 µl CSF were quantitated by qPCR and ELISA, respectively.
(TIF)

Table S1 **Comparison of various CSF parameters (n = 532).**
(DOCX)

Table S2 **Performance of ELISA and PCR assays in Probable and Possible TBM.**
(DOCX)

Table S3 **Performance of Duplex PCR.**
(DOCX)

Table S4 **Odds ratios for the ‘Predictors’ (18 determinants) and ‘new’ tests used in the logistic regression analysis (Probable and Possible TBM patients).**
(DOCX)

Appendix S1 **Proforma for Clinical information.**
(PDF)
