# Supplementary figures and images for: Detection of Mycobacterium tuberculosis GlcB or HspX Antigens or devR DNA Impacts the Rapid Diagnosis of Tuberculous Meningitis in Children
Source: PLoS One. 2012 Sep 12;7(9):e44630. doi: 10.1371/journal.pone.0044630 (PMC3440320; doi:10.1371/journal.pone.0044630)

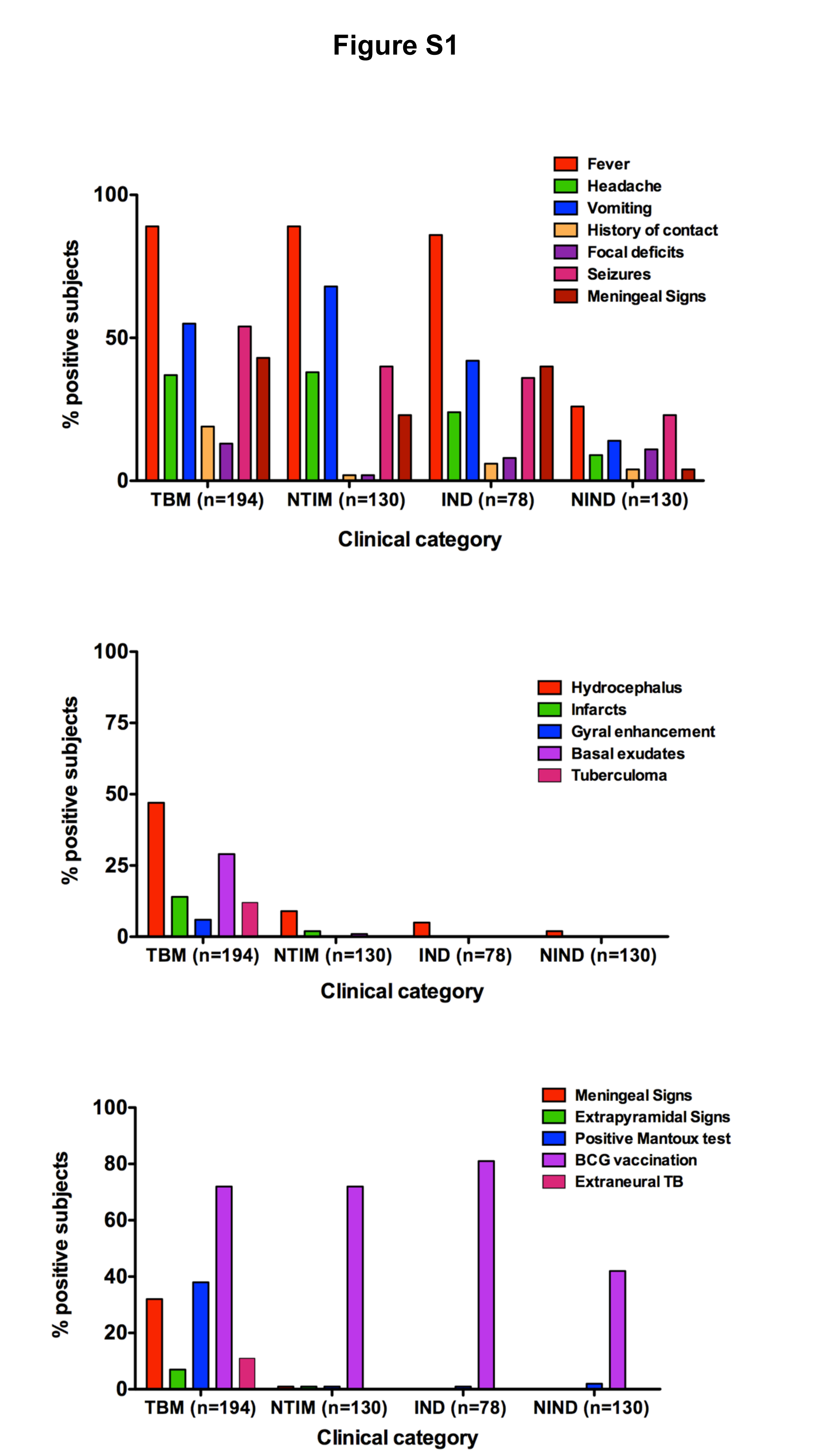

Supplement: Figure S1 — Clinical, radiological and supportive findings. Findings in TBM, NTIM, IND and NIND groups are shown. A. Clinical findings B. Radiological features C. Supportive findings. (TIF) [file pone.0044630.s001.tif]

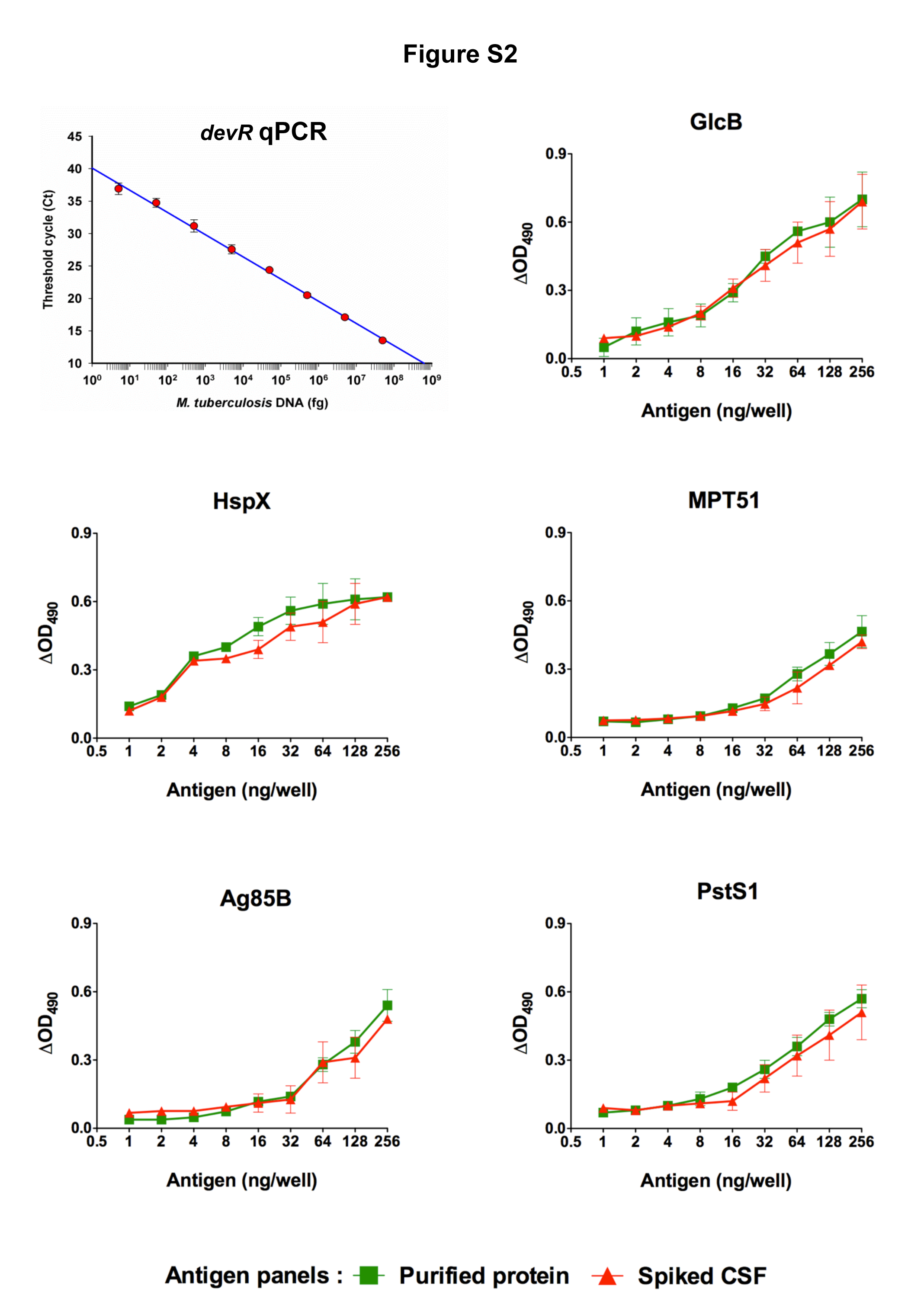

Supplement: Figure S2 — Standard curves for devR qPCR and M. tb antigen ELISAs. devR qPCR standard curve was generated using M. tb DNA over a range of 5 fg to 5×10^7^ fg (from 8 independent experiments, Mean±SD). Ct (threshold cycle) vs. logarithm of the amount of M. tb genomic DNA (fg) at the start of the reaction. ELISA standard curves for M. tb antigens were generated using purified proteins and spiked CSF. ELISA plates were coated with 2-fold serial dilutions of antigen ranging from 256 ng to 1 ng/well. The standard curves were used to quantitate antigen amounts in CSF. (TIF) [file pone.0044630.s002.tif]

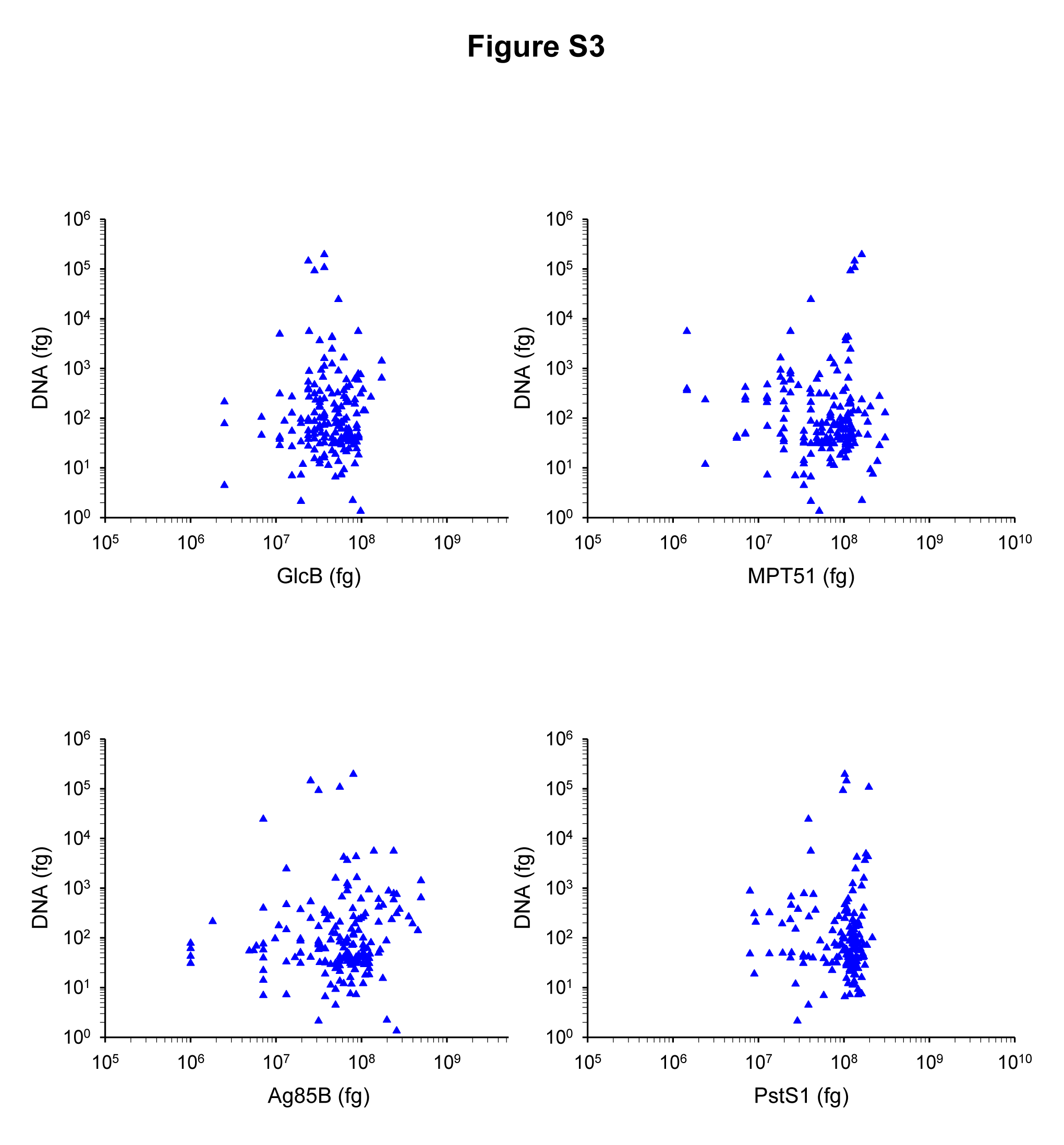

Supplement: Figure S3 — Comparison of M. tb DNA vs. antigen amount in CSF. Graphs for GlcB, MPT51, Ag85B and PstS1 are shown. Starting amounts of DNA and antigen in 5 µl CSF were quantitated by qPCR and ELISA, respectively. (TIF) [file pone.0044630.s003.tif]
