# Supplementary material for: Detection of Mycobacterium tuberculosis GlcB or HspX Antigens or devR DNA Impacts the Rapid Diagnosis of Tuberculous Meningitis in Children
Source: PLoS One. 2012 Sep 12;7(9):e44630. doi: 10.1371/journal.pone.0044630 (PMC3440320; doi:10.1371/journal.pone.0044630)
Supplement: Table S1 — Comparison of various CSF parameters (n = 532). (DOCX) [file pone.0044630.s004.docx]

**Table S1. Comparison of various CSF parameters (n=532)^a^.**

| Parameter | TBM^b^  (n=194) | NTIM  (n=130) | IND  (n=78) | NIND  (n=130) | TBM  *vs.*  NTIM | TBM  *vs.*  IND | TBM *vs.* NIND |
| --- | --- | --- | --- | --- | --- | --- | --- |
| Age (years) | 7  (2.1-11) | 5  (1-10) | 8  (3-13) | 4  (2.1-9) |  |  |  |
| Cells in CSF (per µl) | 50  (10 -150) | 55  (10-250) | 1  (0-5) | 0  (0-0) | p=0.22 | <0.0001 | <0.0001 |
| Number of samples with Cells  (≥10 per µl) | 138 | 91 | 9 | 5 |  |  |  |
| Lymphocyte % | 80  (45-100) | 20  (0-90) | 62.5  (0-100) | 0  (0-0) | <0.0001 | p=0.23 | <0.0001 |
| Number of samples with Lymphocytes (>50%) | 136 | 47 | 39 | 30 |  |  |  |
| Neutrophils | 4  (0-30) | 42.5  (0-90) | 0  (0-0) | 0  (0-0) | <0.0001 | <0.0001 | <0.0001 |
| CSF sugar (mg%) | 42.5  (29-60) | 56.5  (35.5-70) | 72  (56-81) | 68  (60-78) | <0.05  p=0.0015 | <0.0001 | <0.0001 |
| Concomitant Blood Sugar | 96  (80-108) | 101.5  (81-120) | 100  (86-112) | 98  (85-110) | <0.05  p=0.025 | <0.05  p=0.026 | p=0.42 |
| CSF to Blood sugar ratio | 0.44  (0.32-0.59) | 0.56  (0.34-0.69) | 0.67  (0.59-0.81) | 0.71  (0.56-0.78) | <0.05  p=0.046 | <0.0001 | <0.0001 |
| Number of samples with low CSF to Blood sugar ratio  (< 0.5) | 107 | 73 | 22 | 26 |  |  |  |
| CSF protein (mg%) | 98  (69-143) | 62  (33.5-108) | 46  (28-74) | 28  (22-40) | <0.0001 | <0.0001 | <0.0001 |
| Number of samples with raised CSF proteins  (>100 mg/dl) | 93 | 38 | 12 | 7 |  |  |  |

^a^median values are indicated; values in brackets indicate the inter-quartile range.

^b^includes ‘Definite’, ‘Probable’ and ‘Possible’ TBM groups.
