# Supplementary material for: Detection of Mycobacterium tuberculosis GlcB or HspX Antigens or devR DNA Impacts the Rapid Diagnosis of Tuberculous Meningitis in Children
Source: PLoS One. 2012 Sep 12;7(9):e44630. doi: 10.1371/journal.pone.0044630 (PMC3440320; doi:10.1371/journal.pone.0044630)
Supplement: Table S2 — Performance of ELISA and PCR assays in Probable and Possible TBM. (DOCX) [file pone.0044630.s005.docx]

**Table S2. Performance of ELISA and PCR assays in Probable and Possible TBM^a^**.

^a^using cut-offs determined from ROC analysis of ELISA/ qPCR data of ‘Definite’ TBM and NTIM groups (true positives and true negatives, respectively).

^b^based on PCR/ELISA data of ‘Probable and Possible’ TBM and ‘Not-TBM’ group.

| **PCR/ ELISA^b^** | **Sensitivity^c^** | **Specificity^c^** | **PPV^c^** | **NPV^c^** | **LR+** | **LR-** |
| --- | --- | --- | --- | --- | --- | --- |
| **qPCR** | **98**  **(94;99)** | **98**  **(96;99)** | **96**  **(92;98)** | **99**  **(97;99)** | **47**  **(23;98)** | **0.02**  **(0.01;0.07)** |
| **GlcB** | **95**  **(90;97)** | **93**  **(90;95)** | **87**  **(81;90)** | **97**  **(95;98)** | **13**  **(9;19)** | **0.06**  **(0.05;0.14)** |
| **HspX** | **92**  **(87;96)** | **96**  **(94;98)** | **92**  **(87;95)** | **96**  **(93;98)** | **24**  **(14;41)** | **0.08**  **(0;0.27)** |
| **MPT 51** | **94**  **(89;97)** | **96**  **(93;98)** | **92**  **(87;95)** | **97**  **(95;98)** | **22**  **(13;38)** | **0.06**  **(0.03;0.12)** |
| **Ag 85B** | **84**  **(77;89)** | **93**  **(90;96)** | **86**  **(80;91)** | **92**  **(89;94)** | **13**  **(18;19)** | **0.18**  **(0.12;0.25)** |
| **PstS1** | **89**  **(83;93)** | **92**  **(89;95)** | **84**  **(78;89)** | **94**  **(91;96)** | **11**  **(8;16)** | **0.12**  **(0.08;0.18)** |

^c^all values are in percentages, values in brackets denote 95% confidence intervals.
