# Supplementary material for: Detection of Mycobacterium tuberculosis GlcB or HspX Antigens or devR DNA Impacts the Rapid Diagnosis of Tuberculous Meningitis in Children
Source: PLoS One. 2012 Sep 12;7(9):e44630. doi: 10.1371/journal.pone.0044630 (PMC3440320; doi:10.1371/journal.pone.0044630)
Supplement: Table S3 — Performance of Duplex PCR. (DOCX) [file pone.0044630.s006.docx]

**Table S3. Performance of Duplex PCR**.

^a^using data of ‘Definite’ TBM and NTIM groups (true positives and true negatives, respectively).

^b^using data of ‘Probable and Possible’ TBM and Not-TBM groups.

^c^all values are in percentages, values in brackets denote 95% confidence intervals.

| **TBM category** | **Sensitivity^c^** | **Specificity^c^** | **PPV^c^** | **NPV^c^** | **LR+** | **LR-** |
| --- | --- | --- | --- | --- | --- | --- |
| **Definite^a^** | **66**  **(47;80)** | **93**  **(86;96)** | **66**  **(47;80)** | **92**  **(86;96)** | **9**  **(4;16)** | **0.37**  **(0.23;0.62)** |
| **Probable and Possible^b^** | **83**  **(76;88)** | **93**  **(89;95)** | **85**  **(78;89)** | **92**  **(88;94)** | **11**  **(8;16)** | **0.18**  **(0.13;0.26)** |
