# Supplementary material for: Detection of Mycobacterium tuberculosis GlcB or HspX Antigens or devR DNA Impacts the Rapid Diagnosis of Tuberculous Meningitis in Children
Source: PLoS One. 2012 Sep 12;7(9):e44630. doi: 10.1371/journal.pone.0044630 (PMC3440320; doi:10.1371/journal.pone.0044630)
Supplement: Table S4 — Odds ratios for the ‘Predictors’ (18 determinants) and ‘new’ tests used in the logistic regression analysis (Probable and Possible TBM patients). (DOCX) [file pone.0044630.s007.docx]

**Table S4. Odds ratios for the ‘Predictors’ (18 determinants) and ‘new’ tests used in the logistic regression analysis (Probable and Possible TBM patients)^a^.**

| **Parameters^b^/‘new’ tests** | **Predictors** | **Predictors + GlcB** | **Predictors + HspX** | **Predictors + MPT51** | **Predictors + Ag85B** | **Predictors + PstS1** | **Predictors +qPCR** |
| --- | --- | --- | --- | --- | --- | --- | --- |
| ***Clinical criteria*** |  |  |  |  |  |  |  |
| ***Fever > 5 days*** | **2.9 (1.4, 6)** | **1.8 (0.4, 5.6)** | **14.1 (2, 99)** | **5.8 (1.4, 24.2)** | **3.7 (1.3, 10.6)** | **2.2 (0.8, 6)** | **5 (0.5, 43)** |
| ***Headache > 5 days*** | **2.2 (0.4, 4.4)** | **1.8 (0.2, 16)** | **2.4 (0.2, 31.1)** | **1.5 (0.2, 9.2)** | **1.3 (0.3, 5.4)** | **1.3 (0.3, 5.7)** | **4.2 (0.1, 14.5)** |
| *Cough* | 0.7 (0.3, 2) | 0.7 (0.1, 5.4) | 0.2 (0.03, 2.1) | 3.1 (0.6, 16.3) | 1.1 (0.3, 4.5) | 1.3 (0.2, 5.1) | 1.7 (0.2, 2.4) |
| ***Weight Loss*** | **2.9 (5, 4.2)** | **7.9 (1.1, 19.3)** | **8.4 (5.1, 14.1)** | **5.6 (2.8, 15.3)** | **10.2 (1.5, 66.9)** | **8.6 (1.6, 55.9)** | **10.4 (3.1, 12.4)** |
| ***History of contact with TB patient*** | 5.2 (1.8, 15.1) | **10 (1.2, 45.7)** | **4.6 (0.1, 40.4)** | **6.5 (1, 42.6)** | **4.8 (1.1, 20.5)** | **3.9 (0.8, 19)** | **7.7 (2.9, 29.4)** |
| *Focal neurological deficit* | 2.2 (0.7, 6.9) | 1.3 (0.2, 10.8) | 2 (0.1, 30.6) | 3.3 (0.3, 36.4) | 3.4 (0.7, 16) | 2.0 (0.3, 10.9) | 5.8 (1.01, 22.4) |
| *Cranial nerve palsy* | 0.3 (0.06, 1.8) | 0.5 (0.02, 11.8) | 0.02 (0, 0.5) | 0.1 (0, 5.1) | 0.1 (0.01, 1.9) | 0.3 (0.5, 3.9) | 1.6 (0.2, 4.4) |
| *Altered consciousness* | 2.1 (1.04, 4.2) | 1.7 (0.4, 6.2) | 3.5 (0.6, 20.3) | 2.5 (0.6, 11) | 3.1 (1.1, 8.8) | 1.4 (0.5, 3.9) | 1.3 (0.8, 2.4) |
| ***CSF criteria*** |  |  |  |  |  |  |  |
| *Clear appearance of CSF* | 5.4 (1.8, 15.7) | 2.8 (0.3, 28.3) | 4.3 (0.8, 54.3) | 4.3 (0.7, 26.1) | 5.1 (2.4,62.1) | 5.2 (1.2, 22) | 3.3 (0.1, 25.4) |
| ***Cell count > 10-500/μl*** | **3.6 (1.8, 7.26)** | **7.8 (1.7, 36.8)** | **4.4 (0.7, 28.2)** | **4.1 (1, 16.4)** | **5 (1.7, 15)** | **4.4 (1.6, 12.6)** | **5.6 (0.3, 12.4)** |
| ***Lymphocytic predominance > 50%*** | **1.7 (0.8, 3.3)** | **3.3 (0.7, 15.5)** | **12.3 (1.8, 85.4)** | **3.3 (0.8, 13.3)** | **2.9 (1.1, 8.2)** | **2.5 (0.9, 6.38)** | **9.7 (0.7, 13.4)** |
| ***Protein > 100 mg/dl*** | **3.4 (1.6, 7.2)** | **2.2 (0.6, 8.1)** | **3.7 (0.5, 21.9)** | **5 (1.1, 22.4)** | **2.3 (0.8, 6.8)** | **1.2 (0.4, 3.7)** | **3.2 (0.1, 7.4)** |
| ***CSF:Blood sugar ratio <0.5*** | **1.4 (0.6, 2.9)** | **1.4 (0.3, 6.8)** | **0.3 (0.03, 2.7)** | **0.6 (0.1, 3.1)** | **1.1 (0.3, 3.4)** | **1 (0.3, 3)** | **2.1 (0.2, 24.5)** |
| ***Cerebral imaging criteria*** |  |  |  |  |  |  |  |
| ***Hydrocephalus*** | **10.1 (4.3, 23.7)** | **34.3 (5.5,213)** | **70.3 (5.1, 768)** | **9.7 (1.7, 54.5)** | **7.8 (2.2, 28.4)** | **33 (8.5, 127)** | **6.6 (0.3, 26.8)** |
| *Infarcts* | 1.7 (0.2, 14.1) | 1.1 (0.1, 12.9) | 3.6 (1.4, 30.7) | 1.2 (0, 54.6) | 0.3 (0.02, 5) | 0.9 (0.1, 15.2) | 1.4 (0.2, 11.4) |
| ***Basal exudates*** | **11 (4.6, 363.3)** | **32.6 (0.1, 290)** | **24.5 (0.1, 894)** | **16.5 (0.2, 767)** | **13.7 (6.8, 25.5)** | **18 (1, 134.0)** | **6.6 (0.1, 2.4)** |
| *Tuberculoma* | 2.2 (0.3, 16.2) | 2.5 (0.7,5.9) | 4.8 (0.3, 12.5) | 2.8 (0.6, 5.4) | 2.5 (0.9, 7) | 2.6 (1.3, 6.1) | 3 (1.2, 7.5) |
| ***Evidence of TB elsewhere***  ***(extraneural TB)*** | 0.2 (0.02, 14.1) | 1.6 (0.2, 3.7) | 1.3 (1.2, 3.1) | 1.5 (0.3, 5.5) | 1.5 (0.3, 3.3) | 2.4 (0.7, 5.3) | 1.4 (0.4, 3.2) |
| *GlcB* |  | 808.53 (113.7, 5750) |  |  |  |  |  |
| *HspX* |  |  | 750.5 (341.4, 16499) |  |  |  |  |
| *MPT51* |  |  |  | 500.2 (130, 3820.5) |  |  |  |
| *Ag85B* |  |  |  |  | 161.5 (45.5, 572.9) |  |  |
| *PstS1* |  |  |  |  |  | 112.1 (34, 368.5) |  |
| *qPCR* |  |  |  |  |  |  | 987.4 (134.7, 1500) |

^a^Case definition parameters defined by Marais *et al.,* [18]. Values in brackets denote 95% confidence intervals.

^b^Rows in bold and grey shading indicate those parameters (defined in Reference 18) having a significant odds ratio (p<0.05).
