# Supplementary material for: Detection of Mycobacterium tuberculosis GlcB or HspX Antigens or devR DNA Impacts the Rapid Diagnosis of Tuberculous Meningitis in Children
Source: PLoS One. 2012 Sep 12;7(9):e44630. doi: 10.1371/journal.pone.0044630 (PMC3440320; doi:10.1371/journal.pone.0044630)
Supplement: Appendix S1 — Proforma for Clinical information. (PDF) [file pone.0044630.s008.pdf]

Appendix S1. Proforma for Clinical information

Name: **Fathers name:** **Age: Sex:M/F**

Date of admission **C.R.No:** **Date of discharge:**

Postal address:

Phone:

(write “1” in box if the symptom /sign is present, write “0” if not present and enter the duration in space provided)

Those in bold letter are essential entries in all patients

**History/symptoms**	**1 if yes, 0 if No**	**Duration (days)**
**Fever**		
Headache		
**Vomiting**		
Seizures		
Focal seizures		
**Lethargy / irritability**		
**Focal deficit**		
**Sensorial changes**		
Poor feeding		
Cough		
Rapid breathing		
Weight loss		
Skin infection		
Head injury/neurosurgery		
Ear discharge		
Rash		
Type of rash		
Rhinitis		
Constipation		
Immunosupression		
**Measles in last 3 months**		

**History/symptoms**		**1 if yes, 0 if No**	**Duration (days)**
steroid treatment			
Any other predisposing factor			
Any other symptoms:(provide details):			
BCG vaccination			NA
BCG Scar			NA
**Contact with TB patient**			**NA**
Relationship with patient:			
**Prior antibiotic use (IV or oral)**			

Examination findings:

Weight Height: Head circumference:

(write “1” in box if the symptom /sign is present, write “0” if not present)

Those in bold letter are essential entries in all patients

**Finding**	**1 if yes, 0 if No**
Lympadenopathy	
Rash	
Type of rash: (encircle) Petechial /Pupuric /maculopapular /vescicular/other:	
Parotid enlargement	
Skin / mucosal ulcers	
Any other abnormality on Eye / Nose / throat examination…………………………………………………………….	
Any other finding on general physical examination………………………………………………………………..	
Worst GCS:….E…..M……V……	
Bulging Fontanelle	
Decerebrate/decorticate posturing	
**Focal deficits**	
**Extrapyramidal signs**	

Finding	1 if yes, 0 if No
**Cranial Nerve involvement**:	
Details of cranial nerves involved<br>Right 1…….2………3………..4…………5……………. Left 1……2……….3……….4………….5……………	
(encircle)type of deficit: **Chorea, Dystonia, Tremor , Athetosis, Ballismus**<br>**Hemiparesis, Monoparesis, Aphasia, Blindness**	
Chest: Yes/ No, Pneumonia/Effusion/collapse/other………….	
Hepatomegaly	
Spleenomegaly	
Ascites	
Any other significant finding	
New Symptoms and signs during hospital stay and follow up	

Investigations

Gross **CSF**: Clear/ Opalescent/ turbid/ hemorrhagic /cob-web, Pressure- Jet/drops/low

Cell count:

Differential : % lyphocytes: % poly:

CSF Sugar ……… mg/dL, Concomitant Blood Sugar……… mg/dL,

Proteins ……..mg/dL

Montoux test: Positive/negative

PS s/o bacterial ; Y/ N

Blood culture: Positive/negative Organism ……………..

Bacterial/viral/fungal isolation from other sites: Yes/ No.. site………… ………… … …

Blood counts: TLC: DLC:

CT /MRI brain

Hydrocephalous-- Yes/No

Infarcts-- Yes/No

Gyral enhancement- Y/ N

Basal exudates-- Yes/No_____

Tuberculomas-- Yes/No

Number……, Size…..mm X…..mm, margins: regular/irregular, thick wall -Yes/No, dense interior-Yes/No, Midline shift-Yes/No. other features………………..

TB Abscess Yes/No, site………, number …….__________

Cortical enhancement Y/ N

Bacterial Abscess Yes/No, site………, number …________

Extra axial fluid collections Y/ N

Ependymal/meningeal enhancement-- Yes/No___________subarchanoid/subdural

Areas of altered T2 signal……………………………..

CXR abnormalities: Effusion/ Hazy lucencies/ milliary/ cavity/ consolidation/ collapse/ mediastinal LAP/nodular densities/other……………………………….

AFB isolation Extraneural: -- Yes/No, Sputum/Gastric aspirate/LN biopsy/BAL/Pleural fluid/other……….

FNAC/HPE; Y/ N, Tissue……. ….., Finding: Inconclusive/ granulomas/ caseating granuloma/ other………………..

Abdominal X ray/USG/CT abdomen if done: Ascites/LAP/ obstruction/ other………

Any other investigation done to support the diagnosis with findings;

Provisional Diagnosis: TBM/ Pyogenic Meningitis/ Meningoencephalitis/ Seizure disorders/other………………..

Any other investigation:Specific Treatment given Started on Duration

1. ATT- Yes/No ………………………
2. I/V antibiotics ……………………

Response to treatment: Mention features suggestive of good response/poor response

Signs/symptoms		At discharge	1st follow up	2nd follow up
Fever, Vomiting/raised ICP<br>Weight gain/general well being				
Any other				
CSF-<br>TLC/DLC/biochem/…				

…………………………….

Signature of Senior Resident

Name:……………………..
